# ANAT 3.0: a framework for elucidating functional protein subnetworks using graph-theoretic and machine learning approaches

**DOI:** 10.1186/s12859-021-04449-1

**Published:** 2021-10-27

**Authors:** L. F. Signorini, T. Almozlino, R. Sharan

**Affiliations:** 1 Blavatnik School of Computer Science, Tel Aviv University, 6997801 Tel Aviv, Israel; 2 Shmunis School of Biomedicine and Cancer Research, Tel Aviv University, 6997801 Tel Aviv, Israel

**Keywords:** Systems biology, Machine learning, Protein–protein interaction networks, Network biology, Network inference, Interactomics

## Abstract

**Background:**

ANAT is a Cytoscape plugin for the inference of functional protein–protein interaction networks in yeast and human. It is a flexible graphical tool for scientists to explore and elucidate the protein–protein interaction pathways of a process under study.

**Results:**

Here we present ANAT3.0, which comes with updated PPI network databases of 544,455 (human) and 155,504 (yeast) interactions, and a new machine-learning layer for refined network elucidation. Together they improve network reconstruction to more than twofold increase in the quality of reconstructing known signaling pathways from KEGG.

**Conclusions:**

ANAT3.0 includes improved network reconstruction algorithms and more comprehensive protein–protein interaction networks than previous versions. ANAT is available for download on the Cytoscape Appstore and at https://www.cs.tau.ac.il/~bnet/ANAT/.

## Background

Advanced Network Analysis Tool (ANAT) [1, 2] is a graphical, Cytoscape-based [3, 4] tool for the inference of functional protein subnetworks in yeast and human. ANAT allows the user to analyze a process of interest in the context of a protein–protein interaction (PPI) network in four settings: (i) inferring an anchored network that connects a given set of target proteins (e.g., the results of a genome-wide screen to a designated anchor set of proteins that are thought to mediate the process under study); (ii) inferring a general network that connects a given set of proteins to each other; (iii) local exploration of the neighborhood of a given set of proteins, and (iv) finding the shortest paths between pairs of proteins.

ANAT’s main engine is an algorithm to reconstruct subnetworks that link protein nodes of interests (anchors) with target proteins (terminals). It relies on an assembled database of PPI networks, each of which can be viewed as a weighted graph *G* = *(E,V),* where E are the edges, representing PPIs, each of which is assigned with a reliability score, and V are the nodes, representing proteins. It optimizes the reconstruction of a connected subgraph *H(V*_*H*_*,E*_*H*_*)* connecting anchors with terminals by simultaneously optimizing the global weight of the resulting network (sum of weights of the edges in E_H_) and the local length of the shortest path connecting each terminal to one of the anchors. The algorithm is governed by the 0 < α < 0.5 parameter, which balances the local and global criteria. A value closer to 0 emphasizes the local criterion while a value closer to 0.5 emphasizes the global criterion, with α = 0.25, balancing both [2, 5].

Here we present ANAT3.0, an updated version of ANAT that refines the anchored subnetwork reconstruction engine using machine learning techniques. In addition, ANAT3.0 comes with updated PPI networks that integrate KEGG signaling pathways [6] and IntAct interactions [7].

### Implementation

ANAT is implemented as a Java-based plugin for Cytoscape 3.8 and above. The ANAT plugin can be downloaded at http://www.cs.tau.ac.il/~bnet/ANAT/, together with installation instructions, documentation, the user manual and sample inputs and outputs. It is also available on the Cytoscape App Store.

## Materials and methods

For ANAT 3.0, IntAct [7] database interactions were merged with the BioGRID [8] interactions. Since the experimental detection methods (which are used as a feature to assign scores to the final base network in ANAT) for interactions that appeared in both databases were not always concordant, the lowest common ancestral experimental detection method from the PSI-MI ontology was used to represent each such interaction. A total of 109,261 new IntAct interactions (67,864 for human and 41,397 for yeast) were added to the ANAT base PPI network. In addition, we integrated KEGG human and yeast signaling pathways into ANAT base network, by translating the pathways from the KEGG format described in [6] to the ANAT format, by using KEGG proteins as nodes (disregarding small signaling metabolites like PIP3 and decomposing KEGG protein groups into fully connected subnetworks) and protein–protein interactions as edges. All KEGG edges were given a uniform confidence score of 0.6, following consistent rules from [2] of fixed confidence scores for non-experimental data, and the results were merged together with the background network of ANAT, resulting in 2301 new *H. sapiens* interactions and 1003 new *S. cerevisiae* interactions.

The main addition to ANAT's anchored reconstruction algorithm is a machine-learning layer that evaluates candidate proteins predicted by the algorithm and scores them according to their likelihood to appear in the true pathway being sought. To this end, ANAT3.0 exploits known signaling pathways from KEGG. All the nodes (representing KEGG pathway proteins) for each of the 37 (*H. sapiens*) and 20 (*S. cerevisiae*) signaling pathways were concatenated together in a training set and, subsequently, 5 features were obtained for each node as follows: anchors and terminals were extracted individually for each individual pathway by taking KEGG pathway nodes with no inward or no outward edges, respectively. These were used to run ANAT2.0 three times, with three different values of α = {0, 0.25, 0.5}, for every pathway. The output of a single ANAT run contains a list of nodes, each one of them coming with a confidence value, calculated as the percentage of different solutions containing the given node. The first three features of ANAT3.0’s machine learning layer are the confidence values for the given protein, for the three alpha values.

The next set of features represents the proximity of the node to the anchors and terminals, respectively, evaluated using a network propagation calculation [9]. Two network propagations were initialized from anchors and terminals of all pathways, by setting all nodes in the ANAT network to an initial value of 0, and starting nodes with value 1/(number of starting nodes). This yielded two network propagation coefficients for every node in every pathway, which were used as two additional features. If one node is present in more than one KEGG pathway, it will be present twice in the training set, as two different feature vectors. Labels are then assigned to each sample by comparing it to the original KEGG pathways. Finally, eventual KEGG pathway nodes not output by ANAT2.0 are added as an all 0 feature vector with label = 1.

All features were normalized to have mean 0 and variance 1. All nodes vectors were concatenated together to form the input matrix, which was then fed to a random forest classifier using python3's sklearn module with default parameters.

The margin parameter in ANAT2.0 controls the percentage of deviation from the optimum solution to include (a margin of 1.2 will include solutions of 20% deviation from the optimum) [2]. The higher the margin, the bigger the final output network. The aforementioned pipeline was run 6 times, to tune the margin parameter, with 6 different margin values m = {1, 1.2, 1.4, 1.6, 1.8, 2}, and AUC from ROCs and Precision Recall Curves were calculated to select the best model.

The resulting machine learning framework is then applied to a set of features generated for any input of anchors and terminals, and normalized to a standard distribution. The final output of ANAT3.0 is a minimum spanning tree connecting the resulting nodes, each of which is assigned with a confidence score.

## Results and conclusions

In this updated version, which integrates information from BioGrid, IntAct and KEGG, the ANAT human network contains 544,455 interactions and the yeast network has 155,504 interactions. A total of 1026 ANAT2.0 simulations and 114 network propagations were run, for 57 KEGG pathways (37 for *H. sapiens* and 20 for *S. cerevisiae*), with 3 alpha values over 6 margin values m = {1, 1.2, 1.4, 1.6, 1.8, 2}.

5 features were obtained, and for each margin, a random forest classifier was then trained using alternatively (i) all 5 features together (ii) only ANAT2.0’s confidence values (3 features) (iii) only network propagation coefficients from anchors and terminals (2 features), and ROC and precision recall were assessed for all of them with a fivefold cross validation. The performance of a single ANAT2.0 run with the default setting of α = 0.25 was evaluated alongside the new model.

**Fig. 1:**
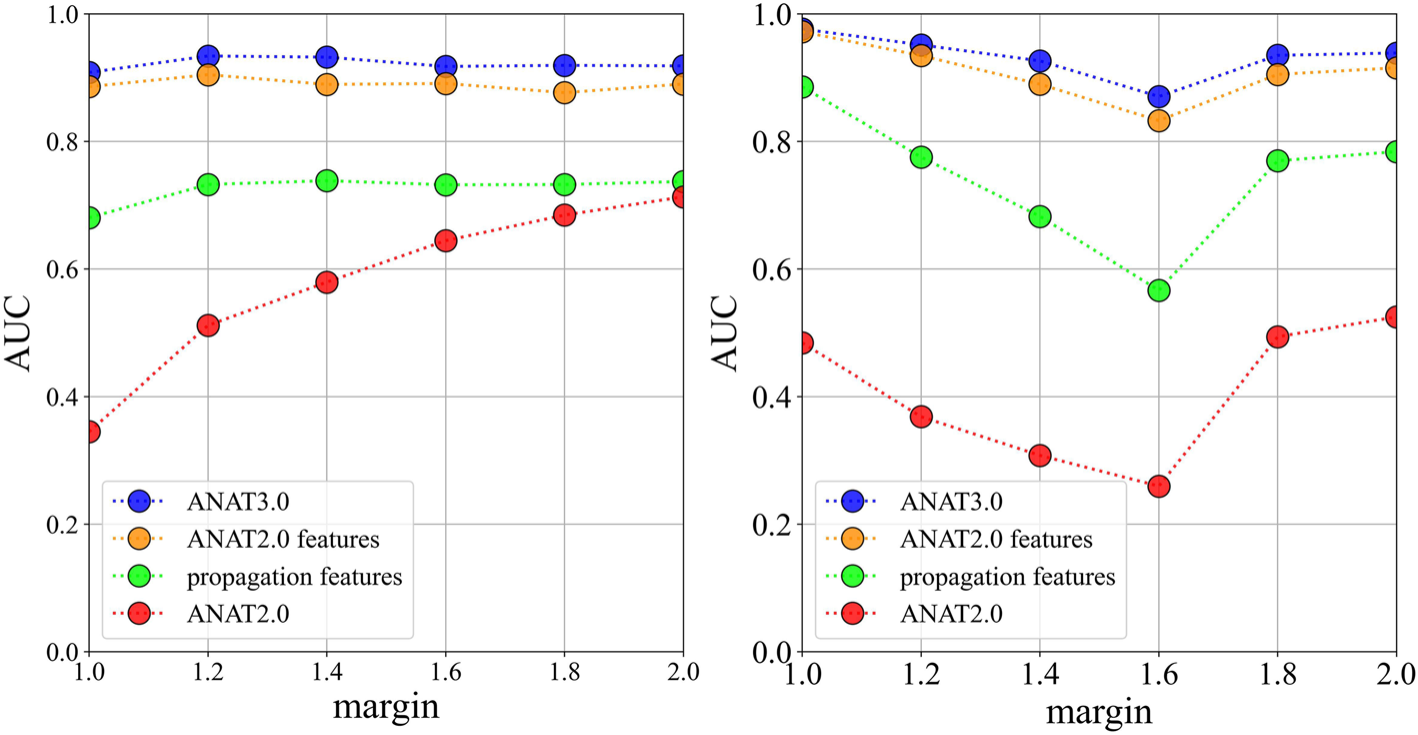
AUC values (*y* axis) for different runs of ANAT3.0, with different sets of features, across ANAT margin values from 1.0 to 2.0 (*x* axis)

The values of AUC for all different setups across different margin values are summarized in Fig. 1. Looking at AUROC and AUPRC values, the margin with the best overall score is m = 1. For m = 1, AUROC for the standard ANAT2.0 was about 0.4, and the AUPRC 0.5. Using network propagation values alone, increased the AUCs to around 0.70 and 0.94, while using all confidence values for the 3 alpha values increased the two AUCs to 0.9, and 0.98, respectively, and using all features together gave a final AUROC of 0.97 and an AUPRC of 0.99. In summary, the new ANAT3.0 classification improved the AUC for both ROC and Precision vs Recall curves from around 0.5 to over 0.95 using a random forest with margin m = 1 and all 5 features, yielding highly accurate predictions.

The machine learning framework can be applied to any given input by generating the five features, normalizing them and applying the trained classifier. The final output of ANAT3.0 is a minimum spanning tree connecting the resulting nodes, each of which is assigned with a confidence score. This yields a refined network structure where nodes are assigned with confidence values using the trained classifier.

### Usage

**Fig. 2:**
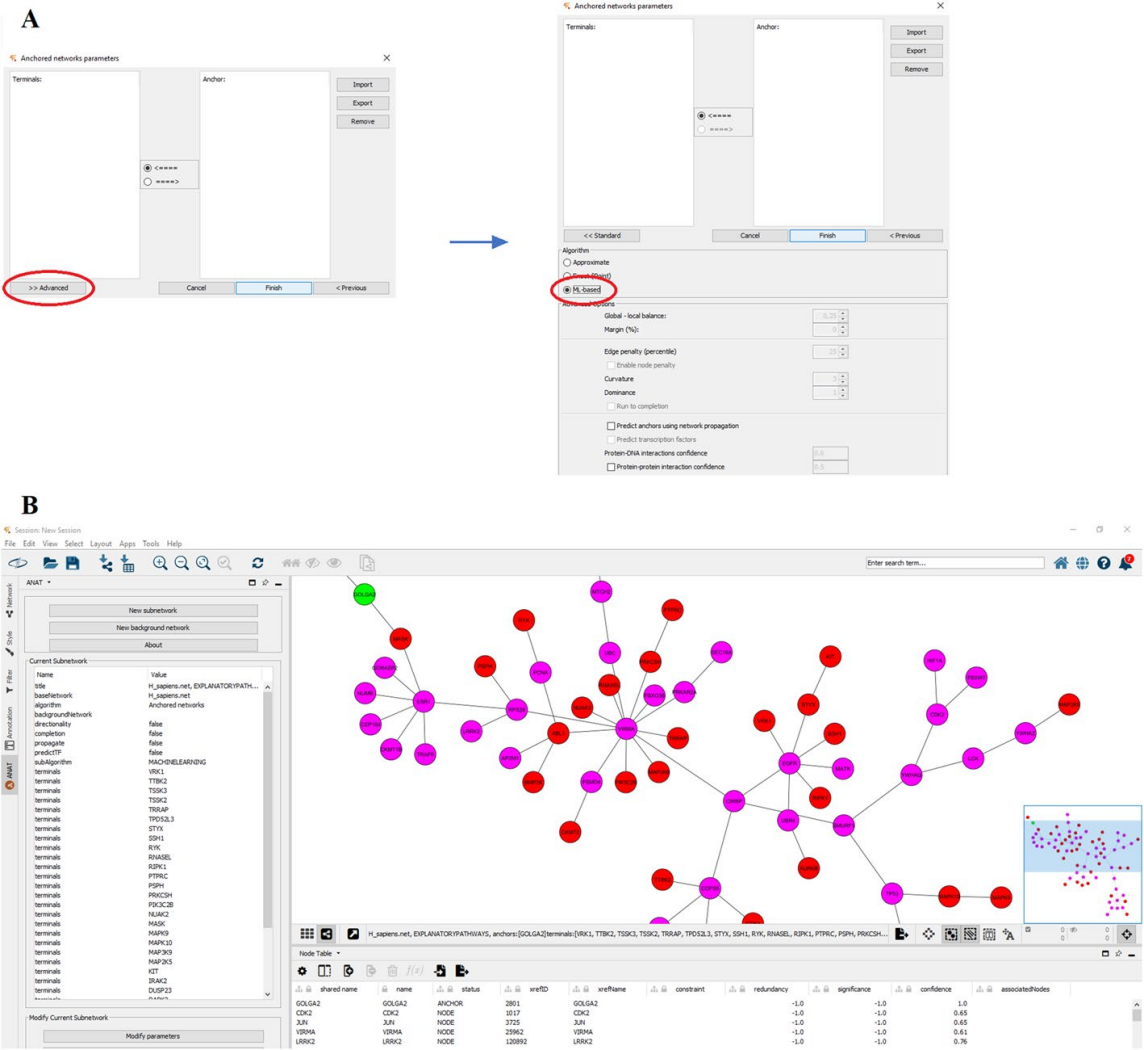
**A** ANAT user interface with highlighted steps to run the machine-learning optimization. **B** ANAT3.0 output example using anchors and terminals from [10]

From the ANAT sidebar on Cytoscape, select “new subnetwork” → “anchored network”, and select the organism of interest (*H. sapiens or S.* cerevisiae), and click “next”. In the next screen, input the names of anchors and terminals, then click on “advanced”, select “ML-based”, and click “finish” (Fig. 2).

### Availability and requirements

Project name: ANAT 3.0. Project home page: http://www.cs.tau.ac.il/~bnet/ANAT/. Operating system(s): Operates on all major operating systems. Programming language: Java, Python3. Other requirements: Cytoscape 3.8 and above. License: Free for academic use. Any restrictions to use by non-academics: License needed.

## Data Availability

KEGG pathway data is available at https://www.genome.jp/kegg/. IntAct interaction data is available at https://www.ebi.ac.uk/intact/.

## Abbreviations

AUC: Area under the curve.
AUPRC: Area under the precision vs recall curve.
AUROC: Area under the receiver operating curve.
PPI: Protein–protein interaction
